# EXPERT CONSENSUS PANEL RECOMMENDATIONS FOR MEDICAL AND MENTAL HEALTH CARE IN ASSISTED LIVING

**DOI:** 10.1093/geroni/igac059.1115

**Published:** 2022-12-20

**Authors:** Sheryl Zimmerman, Philip Sloane, Christopher Wretman, Kevin Cao, Johanna Silbersack, Paula Carder, Kali Thomas, Suzanne Meeks

**Affiliations:** University of North Carolina, Chapel Hill, North Carolina, United States; University of North Carolina-Chapel Hill, Chapel Hill, North Carolina, United States; University of North Carolina at Chapel Hill School, Chapel Hill, North Carolina, United States; University of Illinois, Chicago, Illinois, United States; University of North Carolina, Chapel Hill, North Carolina, United States; Portland State University, Portland, Oregon, United States; Brown University, Providence, Rhode Island, United States; University of Louisville, Louisville, Kentucky, United States

## Abstract

Assisted living (AL) is now the largest residential provider of long-term care in the U.S., and the acuity of AL residents has been rising. However, AL is not a health care setting, and concern has grown about the medical and mental health care needs of AL residents. Because there is no guidance to inform this care, a Delphi consensus panel of 19 experts was convened, and their recommendations were compared to actual practice. Panelists rated 200 discrete items, 43 of which met standard recommendation cut points (rated as important by at least 75% of panelists) and reflected four critical components of AL: the tenets of AL, the role AL plays in providing long-term care for persons with dementia, the need for pragmatism in response to the diversity of AL, and workforce needs. Next steps regarding implementation of the recommendations will be discussed.

